# Focal neuroendocrine carcinoma mixed with adenocarcinoma of the gallbladder with aggressive lymph node metastasis in a patient who did not meet the mixed neuroendocrine–non-neuroendocrine neoplasm criteria

**DOI:** 10.1007/s12328-021-01547-8

**Published:** 2021-11-12

**Authors:** Yoshie Kadota, Shinsuke Funakoshi, Shigemichi Hirose, Eisuke Shiomi, Masanori Odaira, Haruka Yagishita, Yosuke Kobayashi, Fumiki Toriumi, Seiichi Tamai, Takashi Endo, Hirohisa Harada

**Affiliations:** 1 Department of General and Gastrointestinal Surgery, Tokyo Saiseikai Central Hospital, 1-4-17 Mita, Minato-ku, Tokyo, 108-0073 Japan; 2 Department of Surgery, Nippon Koukan Hospital, 1-2-1 Kokandori, Kawasaki Ward, Kawasaki, Kanagawa 210-0852 Japan; 3 Department of Medical Oncology, Tokyo Saiseikai Central Hospital, 1-4-17 Mita, Minato-ku, Tokyo, 108-0073 Japan; 4 Department of Diagnostic Pathology, Tokyo Saiseikai Central Hospital, 1-4-17 Mita, Minato-ku, Tokyo, 108-0073 Japan; 5 Department of Radiology, Tokyo Saiseikai Central Hospital, 1-4-17 Mita, Minato-ku, Tokyo, 108-0073 Japan

**Keywords:** Gallbladder, Adenocarcinoma, Neuroendocrine carcinoma, Mixed adenoneuroendocrine carcinoma (MANEC), Mixed neuroendocrine–non-neuroendocrine neoplasm (MiNEN), Lymph node metastasis

## Abstract

A 70-year-old Japanese woman who was treated for interstitial pneumonia (IP) with steroid therapy developed cholecystitis. A serial computed-tomography (CT) imaging showed irregular thickness of the fundus wall of the gallbladder and two rapidly enlarged lymph nodes (LNs): number (no.) 12 and no. 8a. Positron-emission tomography-computed tomography (PET-CT) scan showed an abnormal uptake at the site of the gallbladder tumor and those LNs. We subsequently performed open radical cholecystectomy and LN dissection of the no. 12 and 8a LNs, following complete remission of IP. The histology showed gallbladder adenocarcinoma, with a single focus of neuroendocrine carcinoma (NEC) component of less than 30%; Ki-67 index > 80%, synaptophysin (Syn) (+), chromogranin A (CgA) (+), and clusters of differentiation (CD) 56 (+) (T2bN1M0, Stage IIIB). LN no. 8a was diffusely metastatic with NEC components. LN no. 12c, which was adjacent to the cystic duct, revealed necrosis without apparent tumor cells, but was highly suspicious for tumor necrosis. The final diagnosis was adenocarcinoma of the gallbladder with focal NEC (< 30%), which did not meet the criteria for mixed neuroendocrine–non-neuroendocrine neoplasm (MiNEN). Postoperatively, she completed 4 cycles of adjuvant chemotherapy for NEC (Cisplatin plus Etoposide), and no recurrence was observed after 12 months.

**Supplementary Information:**

The online version contains supplementary material available at 10.1007/s12328-021-01547-8.

## Introduction

The occurrence of gallbladder neuroendocrine carcinoma (NEC) is very rare, which is reported to be 2.2% of all gallbladder cancers, which usually presents with advanced stage, lymph nodes (LNs) metastases, and poor prognosis [1]. More than a third of gallbladder NEC is mixed with a non-neuroendocrine component, most frequently, adenocarcinoma [2, 3]. According to the 2019 World Health Organization (WHO) Classification, these mixed tumors are called mixed neuroendocrine–non-neuroendocrine neoplasm (MiNEN) (previously known as MANEC: mixed adenoneuroendocrine carcinoma), if both components are present in more than 30% of the tumor [4]. In addition, neuroendocrine tumor (NET) Grade (G) 3 and NEC are categorized separately in the new classification. Well-differentiated NET is now NET G3, and poorly differentiated NET is considered NEC, both with a mitotic rate (mitoses/2 mm^2^) > 20, or Ki-67 > 20% [4]. The median overall survival of biliary NEC and MiNEN are reported as 9.6 and 12.2 months, respectively [5]. Because gallbladder MiNEN is a rare tumor, the overall survival has not been well investigated. Furthermore, the true nature of how aggressive the tumor progresses have not been well reported. In this case report, while the patient was treated for interstitial pneumonia (IP), we were able to follow the rapid progression of the gallbladder adenocarcinoma with focal NEC (< 30%) and LN metastasis, over the course of 4 months with serial computed-tomography (CT) imaging. This was a unique case where we were able to observe the behavior of this aggressive tumor, while waiting for the complete remission of IP before performing surgical intervention.

## Case report

A 70-year-old woman was referred to our surgical division by her pulmonologist who was treating her for idiopathic IP for 1 year prior to presentation, for an incidental finding of chronic cholecystitis found on the CT scan. Three months prior to referral, she was started on steroid therapy for exacerbation of her IP, which was tapered down to Prednisolone (PSL) 12.5 mg/day at the time of consultation. The patient reported recurring mild episodes of right subcostal pain, which she did not seek medical attention for. Her past medical history was unremarkable except for childhood asthma and untreated uterine myoma. Most notably, her past surgical history was significant for endoscopic mucosal resection (EMR) for a 4 mm NET G1 of the lower rectum, 20 months prior to initial consultation. Other past surgical history included an appendectomy for appendicitis in childhood, and a left partial oophorectomy for two benign ovarian cysts. She was not taking any medications except for PSL 12.5 mg, which was tapered down over 1 month from the initial dosing of 30 mg. Her family history was non-contributory. She reported no known drug allergies. Her social history included smoking one pack (20 cigarettes) per day for 40 years, which she recently quit, but she denied drinking alcohol or use of recreational drugs. She is a retired desk worker. Laboratory data were within normal limits as shown in Table S1, including carcinoembryonic antigen (CEA) and cancer antigen 19-9 (CA19-9).

**Fig. 1:**
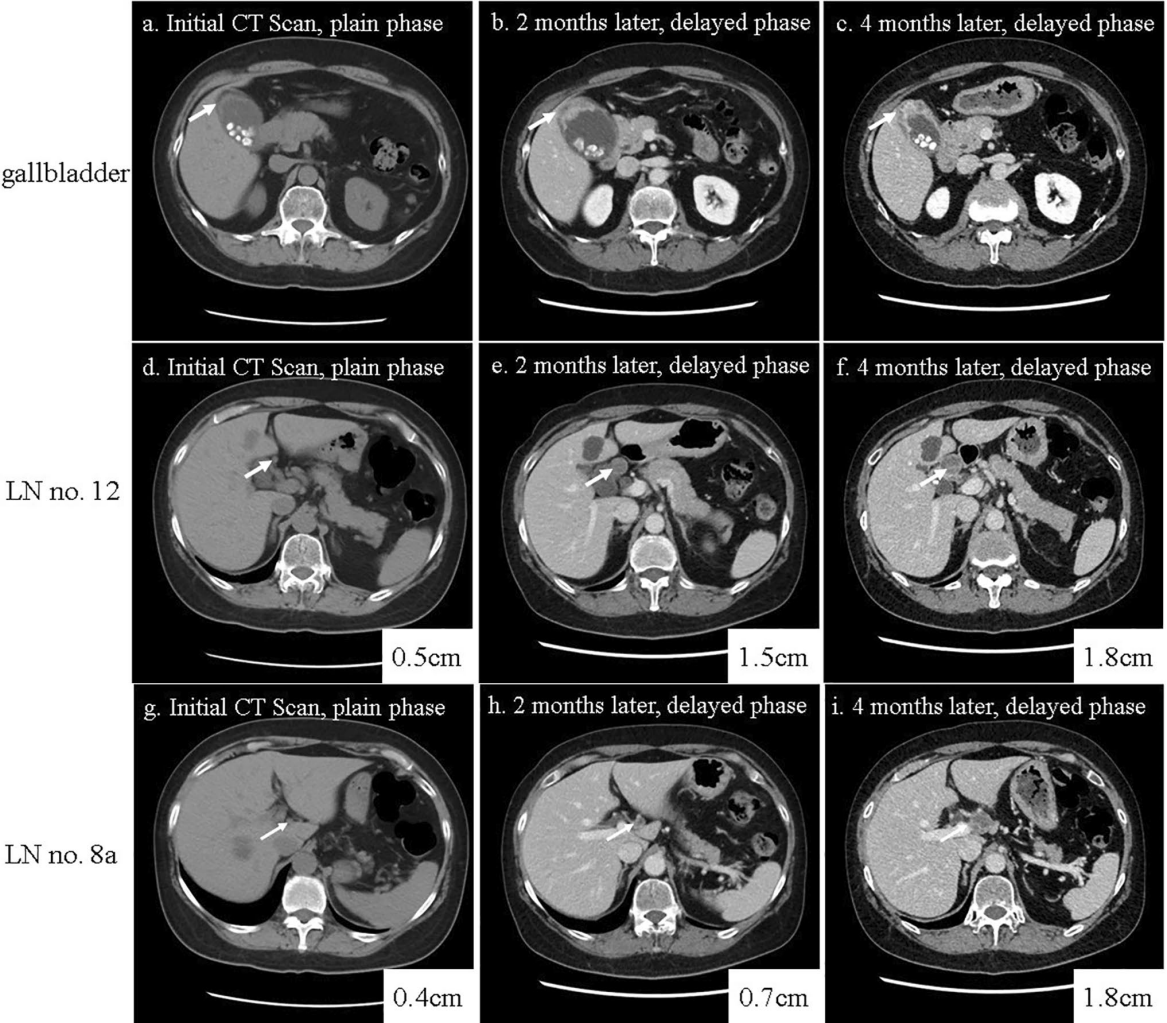
Preoperative serial CT scans of the gallbladder. The figure shows preoperative serial CT scans showing rapid tumor growth in the gallbladder and the adjacent LNs, over the course of 4 months. **a, d, g** CT scan at the time of surgical consultation. **b, e, h** Follow-up CT scan, 2 months later. **c, f, i** Follow-up CT scan, 4 months later. Initial CT scan showed no apparent tumors in the gallbladder or the LNs (**a, d, g**). CT scan at 2 and 4 months revealed gradual thickening of the fundus wall of the gallbladder (**b, c**), rapid growth of the LN no. 12 (**e, f**), and the LN no. 8a (**h, i**). No apparent liver infiltration or peritoneal dissemination were observed

**Fig. 2:**
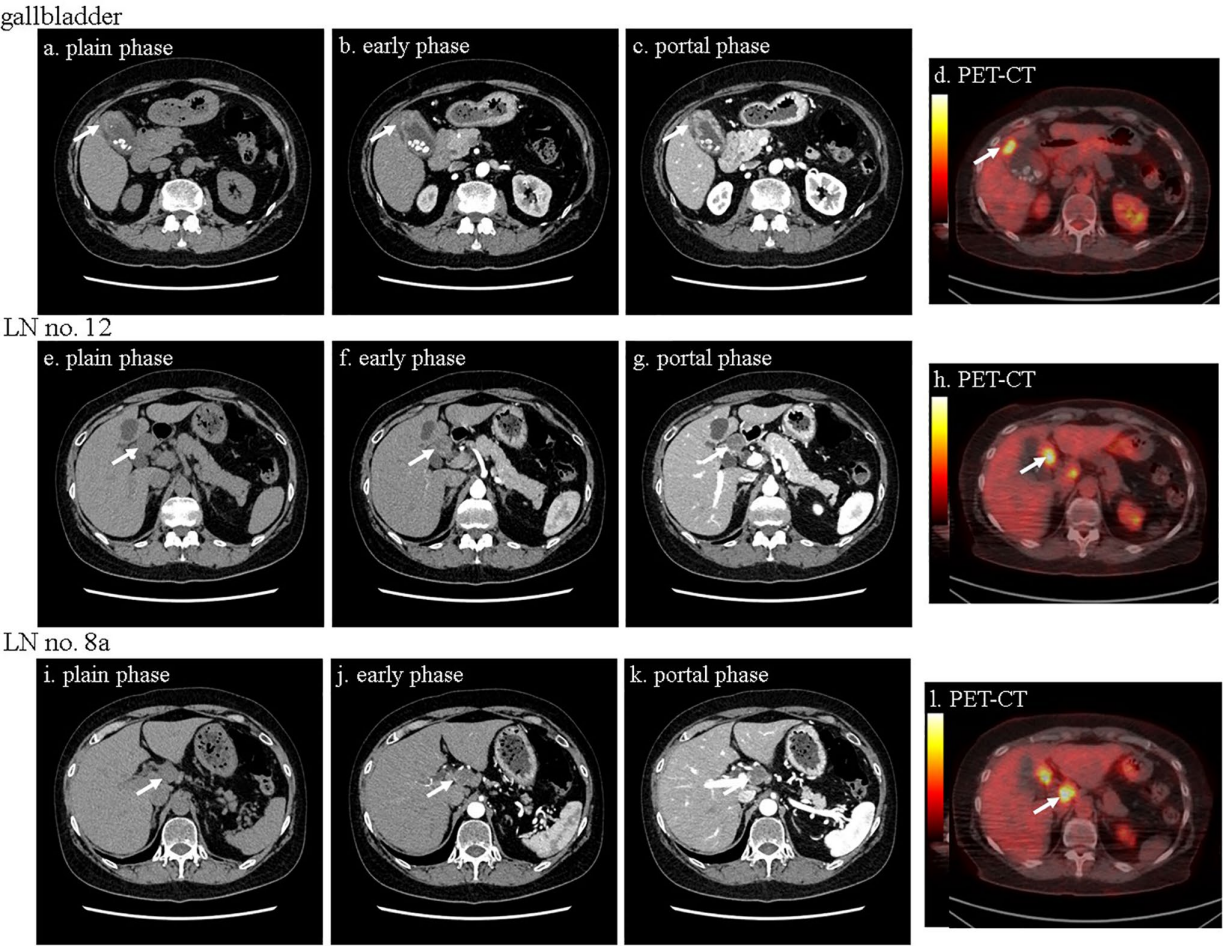
Dynamic CT scan and PET-CT scan findings of the gallbladder. The figure shows dynamic CT scan and PET-CT scan results, 4 months later, taken at the same time as Fig. 1c, f, and i, preoperatively. The irregularly thickened wall showed an increased density in the fundus of the gallbladder on CT scan (**a–c**). In the LNs No. 12 and 8a, the cortex of the LNs were slightly enhanced, with non-homogenous contrast enhancement seen within both LNs (**e–g, i–k**). PET-CT scan showed increased ^18^F-FDG uptake in the fundus of the gallbladder, LNs no. 12, and no. 8a (**d, h, l**)

Initial CT showed signs of cholecystitis with a mild and diffuse wall thickening with multiple small gall stones (Fig. 1). A follow-up CT scan, which was performed 2 months later, revealed irregular thickening of the gallbladder fundus wall in addition to two enlarged LNs; number (no.) 12 and no.8a, 1.5 cm and 0.7 cm, respectively. Even though malignancy was suspected at this point, because she was still on 10 mg of PSL, surgical intervention was postponed. Around the same time, she was treated with a single dose of 50 mg of Azathioprine by her pulmonologist. Subsequent CT scan at 4 months showed persistent irregular wall thickening of the gallbladder fundus. Furthermore, no. 12 LN increased in its size to 1.8 cm from 1.5 cm, and no. 8a LN increased in its size to 1.8 cm from 0.7 cm. Both LNs emerged and enlarged rapidly, presenting with non-homogenous low densities within the LNs, suspicious for necrosis (Fig. 1). On the Positron-emission tomography-computed tomography (PET-CT) scan, an abnormal uptake of fluorodeoxyglucose (^18^F-FDG) were observed in the fundus of the gallbladder, as well as no. 12 and no. 8a LNs (Fig. 2). In addition, T1-, T2-, and diffusion-weighted magnetic resonance imaging (MRI) was performed. The findings were notable for high intensity observed in the fundus of the gallbladder, as well as LNs no. 12 and no. 8a (Fig. S1).

Serial CT scan, MRI, and PET-CT scan all pointed to the diagnosis of the gallbladder tumor with LN metastases. She underwent radical cholecystectomy with liver-wedge resection and LN dissection, 5 months after the initial surgical consultation. The LN dissection included all hot spots seen on the PET-CT scan, including the no. 12, no. 8a, and no. 13a lymphatic basins. No liver metastases or peritoneal dissemination were found. Intraoperative ultrasound showed no infiltration to the liver bed, and therefore, no further hepatectomy was recommended. Careful excision of the enlarged LN no. 8a revealed significantly fragile LN, indicating potential metastasis. Intraoperative frozen section of the cystic duct was negative for tumor cells. The postoperative course was uneventful except for mildly symptomatic delayed gastric emptying, which is not unusual in patients who are treated with hepatoduodenal ligament dissection. She was discharged home from the hospital on postoperative day 17.

**Fig. 3:**
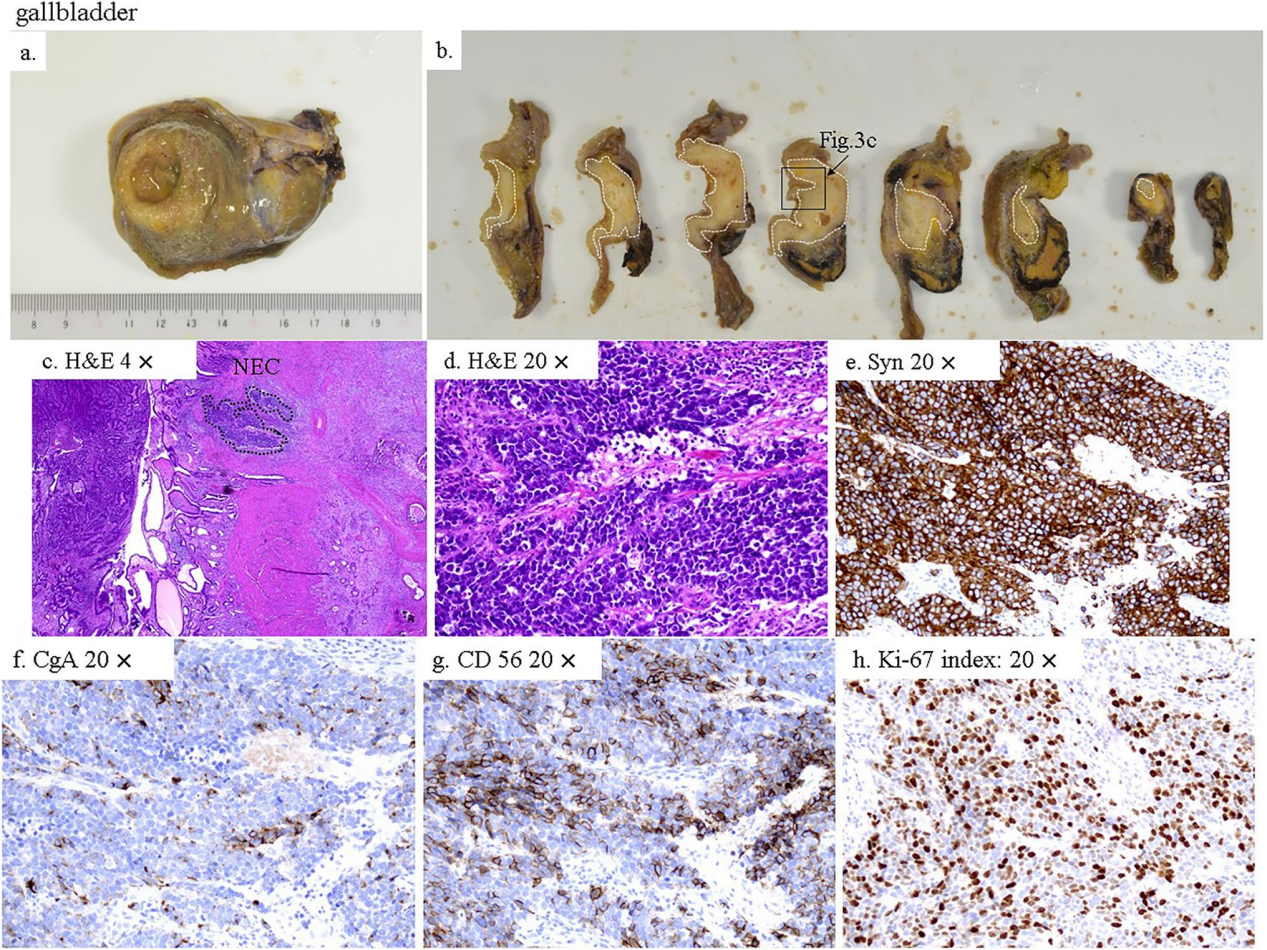
Histopathological analysis of the gallbladder. **a** the gross specimen of the surgically excised gallbladder. **b** axial sections of the gallbladder shown in **a**. The neoplasm in the fundus was diagnosed as well-to-moderately differentiated adenocarcinoma with focal NEC components (**c, d**), which were diffusely positive for Syn (**e**), while CgA (**f**) and CD56 (**g**) were focally positive, and Ki-67 was over 80% (**h**) (T2bN1M0, Stage IIIB, according to the UICC 8th edition). *H&E* hematoxylin and eosin staining, *Syn* synaptophysin, *CgA* chromogranin A, *CD 56* clusters of differentiation, *NEC* neuroendocrine carcinoma, *× 20* × 20 magnification

**Fig. 4:**
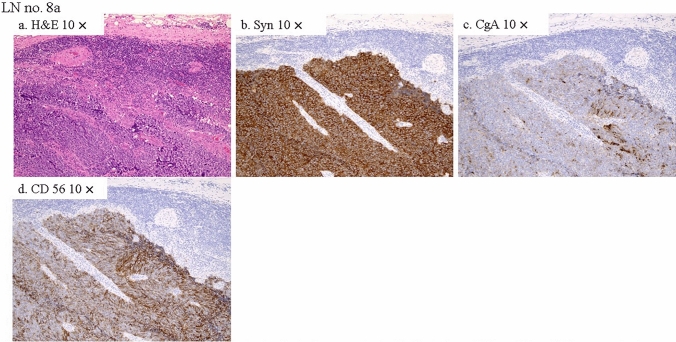
Histopathological analysis of the LN 8a. Metastases were only detected in LN No. 8a (**a**) which was completely occupied by highly proliferative NEC cells, which were positive for Syn (**b**), CgA (**c**), and CD 56 (**d**), same as the gallbladder NEC components (Fig. 3**e–g**). *H&E* hematoxylin and eosin staining, *Syn* synaptophysin, *CgA* chromogranin A, *CD 56* clusters of differentiation, *NEC* neuroendocrine carcinoma, *× 10* × 10 magnification

Histology of the gallbladder showed well-to-moderately differentiated adenocarcinoma, according to the UICC 8th edition), with a focal NEC component (< 30%), which was positive for synaptophysin (Syn), chromogranin A (CgA), and clusters of differentiation (CD) 56, and Ki-67 index > 80% on immunohistochemistry (T2bN1M0 Stage IIIB, according to the UICC 8th edition) (Fig. 3). Based on these findings, the tumor was diagnosed as gallbladder adenocarcinoma with NEC, which did not meet the criteria for MiNEN. LN no. 8a was diffusely metastatic with NEC components, which were positive for Syn, CgA, and CD 56, and was the only LN that showed clear metastasis based on histology (Fig. 4). The LN no. 12c, which was adjacent to the cystic duct was predominantly necrotic without apparent tumor cells, but was highly suspicious for tumor necrosis. Because the histology of the LN revealed diffusely metastatic NEC, she received 4 cycles of adjuvant chemotherapy with Cisplatin (80 mg/m^2^) on day 1 and Etoposide (100 mg/m^2^) on days 1–3, every 4 weeks, beginning 1 month postoperatively. No recurrence was observed for 12 months, at the time of this study.

## Discussion

Gallbladder MiNENs are extremely rare. Costa et al. [6] published a literature review in 2021, and reported that there are only 24 cases of gallbladder MiNEN (which used to be called MANEC) including this case, reported across the literature. Although our case did not meet the criteria for MiNEN [4], this report supports our understanding of how rare and aggressive the gallbladder MiNEN is, and therefore, close postoperative surveillance is critical.

In MiNEN, NEC components are known to show more aggressive patterns [3, 7]. Generally, in MiNEN, aggressiveness and prognosis are driven by the most aggressive neoplastic components [8, 7]. Furthermore, MiNEN with predominant NEC components are more likely to metastasize to LNs [3]. Several authors reported that patients with resectable cases of MiNEN who underwent complete surgical resection had overall better prognosis [2, 8, 9]. Chen et al. [1] described the aggressive nature of the gallbladder NEC, and reported that patients with NEC had significantly higher rate of LN metastases and poorer prognosis, when compared to patients with gallbladder adenocarcinoma. In their study, median survival time was found to be longer in patients who underwent surgical resection, chemotherapy, and radiation therapy, when compared to patients who received palliative resection of the gallbladder cancer.

This is a unique case report where we were able to observe the behavior of this aggressive tumor, while waiting for the complete remission of IP before performing surgical intervention, which took place 5 months after the initial diagnosis of cholecystitis. Consequently, the serial CT scan demonstrated the tumor rapidly emerging and progressing in just 4 months, especially in the no. 8a LN.

Glucocorticoid PSL is known for its anti-inflammatory response in many chronic inflammatory diseases. It is also widely used as part of various cancer treatments. In our patient, the oral PSL and the single dose Azathioprine administration may have contributed to the rapid progression of the gallbladder adenocarcinoma with NEC. However, the effects of immunosuppressive therapies on the tumor progression remain unknown [10]. Further studies are warranted.

If neoadjuvant chemotherapy is considered, an endoscopic-ultrasonography-guided-fine-needle aspiration (EUS-FNA) should be considered unless there are no contraindications. However, in hindsight, EUS-FNA likely would have not yielded the correct diagnosis in this patient, because the gallbladder NEC was small. Furthermore, majority of the providers biopsy one representing LN to rule out metastasis. In this patient, unless LN no. 8a was selected over LN no. 12c by chance, NEC components may have been missed.

Okada et al. [11] reported a similar case of an aggressive focal gallbladder NEC mixed with adenocarcinoma which did not meet the criteria for MiNEN. In their report, the patient was surgically treated with subsequent adjuvant chemotherapy, successfully. However, 4 months later, the patient presented with multiple metastases of NEC in the para-aortic LNs. Despite undergoing a second round of chemotherapy, the patient expired 8 months after the initial surgery. These cases highlight how aggressive the NEC components behave, regardless of the size of the primary NEC. Due to the aggressive nature of NEC components with frequent LN involvement, we highly recommend performing the standard recommended dissection of the hepatoduodenal ligament, no. 12, no. 8a, and no. 13a LNs, based on our experience combined with other previous reports [1, 3, 5, 11].

There is currently no consensus on the postoperative adjuvant chemotherapy, but it is usually determined based on the most aggressive components of MiNEN [8, 7, 12]. The latest National Comprehensive Cancer Network (NCCN) guideline recommends several therapy options for resectable gallbladder cancer, including; (a) observation without any additional therapy, (b) neoadjuvant/adjuvant systemic chemotherapy, and (c) fluoropyrimidine + radiotherapy (RT) [13]. On the other hand, NCCN guideline recommends several therapy options for resectable extrapulmonary NEC, including (a) resection + adjuvant chemotherapy + / − RT, (b) neoadjuvant chemotherapy + / − RT + resection, (c) chemotherapy alone, (d) RT alone, and (e) definitive chemoradiation with Cisplatin + Etoposide (or Carboplatin + Etoposide) [14]. In our patient, the most aggressive component was determined to be NEC, as supported by LN metastases. Therefore, the patient subsequently received 4 cycles of Cisplatin (80 mg/m^2^) on day 1 and Etoposide (100 mg/m^2^) on days 1–3, every 4 weeks.

At the time of this study which was 12 months after total cholecystectomy with LN dissection, no recurrence of the tumor was observed. If the predominant component of the MiNEN is NEC, the tumor tends to behave more aggressively, presenting with frequent LN metastases [3, 7]. Gallbladder NEC is notorious for high recurrence rate, poor morbidity, and mortality [1, 5, 11, 12]. The patient will continue to be closely monitored.

This patient underwent EMR for a lower rectum G1 NET, 2 years prior to this procedure. The previous tumor was a different tumor, which was negative for Syn, CgA, CD56, and Ki-67 on immunohistochemistry, in contrast to NEC components of this tumor.

Although each component of MiNEN must account for at least 30% of the whole neoplasm to meet the criteria for MiNEN, several authors suggested that the prognosis was influenced by the most aggressive histological components, regardless of the percentage of those components [8, 9, 15]. Our patient had less than 30% of NEC components, who did not meet the criteria for MiNEN, but presented with diffusely metastatic LNs with NEC components. Because NEC components were the most aggressive histological components, our patient was successfully treated with adjuvant chemotherapy for NEC, with no recurrence observed at 12 months follow-up, at the time of this study. Our patient may raise a question challenging the current 30% cut off as defined by the WHO 5th edition. In our patient, the tumor had less than 30% of NEC components which did not satisfy the criteria for MiNEN, but behaved very aggressively with early LN metastases. Therefore, aggressive treatment including surgical intervention and adjuvant chemotherapy may be necessary for patients presenting with any percentage (< 30%) of NEC components that behaves like MiNEN (> 30%).

In conclusion, our case highlights the fact that the gallbladder adenocarcinoma with NEC (< 30%) which did not meet the criteria for MiNEN, may rapidly progress in just a few months with LN metastases, driven by NEC components. Radical surgery combined with adjuvant chemotherapy (4 cycles of Cisplatin with Etoposide) showed good results over this highly aggressive tumor. Based on our limited experience, for patients with gallbladder adenocarcinoma with NEC, we highly recommend performing cholecystectomy with further LN dissection of the LNs no. 12, no. 8a, and no. 13a, even if it does not meet the criteria for MiNEN. Further investigation is warranted.

## Supplementary Information

Below is the link to the electronic supplementary material.

Supplementary file1 Gadolinium ethoxybenzyl diethylenetriamine penta-acetic acid (Gd-EOB-DTPA)-enhanced MRI findings of the gallbladder and diffusion-weighted MRI findings of the LNs No. 12 and 8a. (JPG 208 KB)

Supplementary file2 Fig. S1 shows dynamic EOB-MRI results at 2 weeks prior to surgery. The irregularly thickened fundus wall of the gallbladder showed iso- to low-intensity in the fat suppressed T1-weighted image (a), and low-intensity in the T2-weighted image (b). Diffusion-weighted image of the gallbladder showed hyper intensity (c), while both enlarged LNs, no. 12 and 8a (d), showed high-intensity area, surrounding a central low-intensity area, suggestive of central necrosis. Dynamic MRI showed increased intensity in the fundus of the gallbladder (e-h), enlarged in Fig. S1g. (JPG 158 KB)
