# Supplementary figures and images for: Focal neuroendocrine carcinoma mixed with adenocarcinoma of the gallbladder with aggressive lymph node metastasis in a patient who did not meet the mixed neuroendocrine–non-neuroendocrine neoplasm criteria
Source: Clin J Gastroenterol. 2021 Nov 12;15(1):185–91. doi: 10.1007/s12328-021-01547-8 (PMC8858271; doi:10.1007/s12328-021-01547-8)

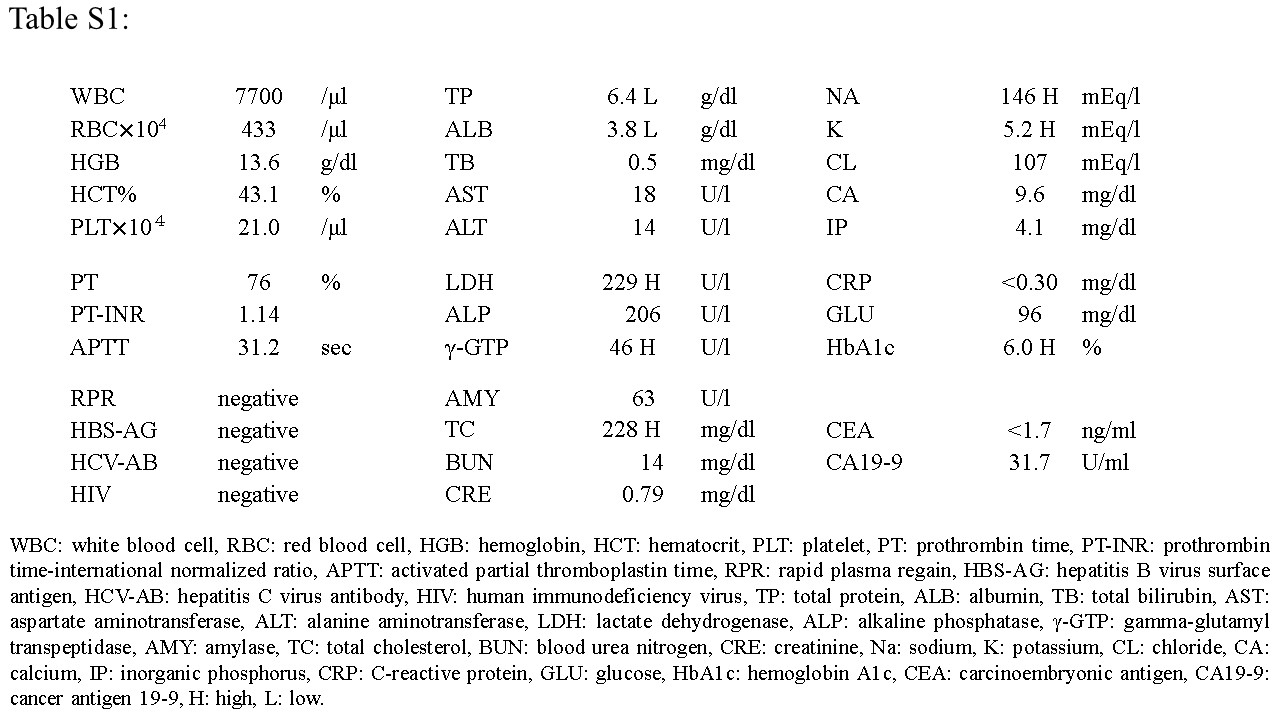

Supplement: Supplementary file 1 — Supplementary file1 Gadolinium ethoxybenzyl diethylenetriamine penta-acetic acid (Gd-EOB-DTPA)-enhanced MRI findings of the gallbladder and diffusion-weighted MRI findings of the LNs No. 12 and 8a. (JPG 208 KB) [file 12328_2021_1547_MOESM1_ESM.jpg]

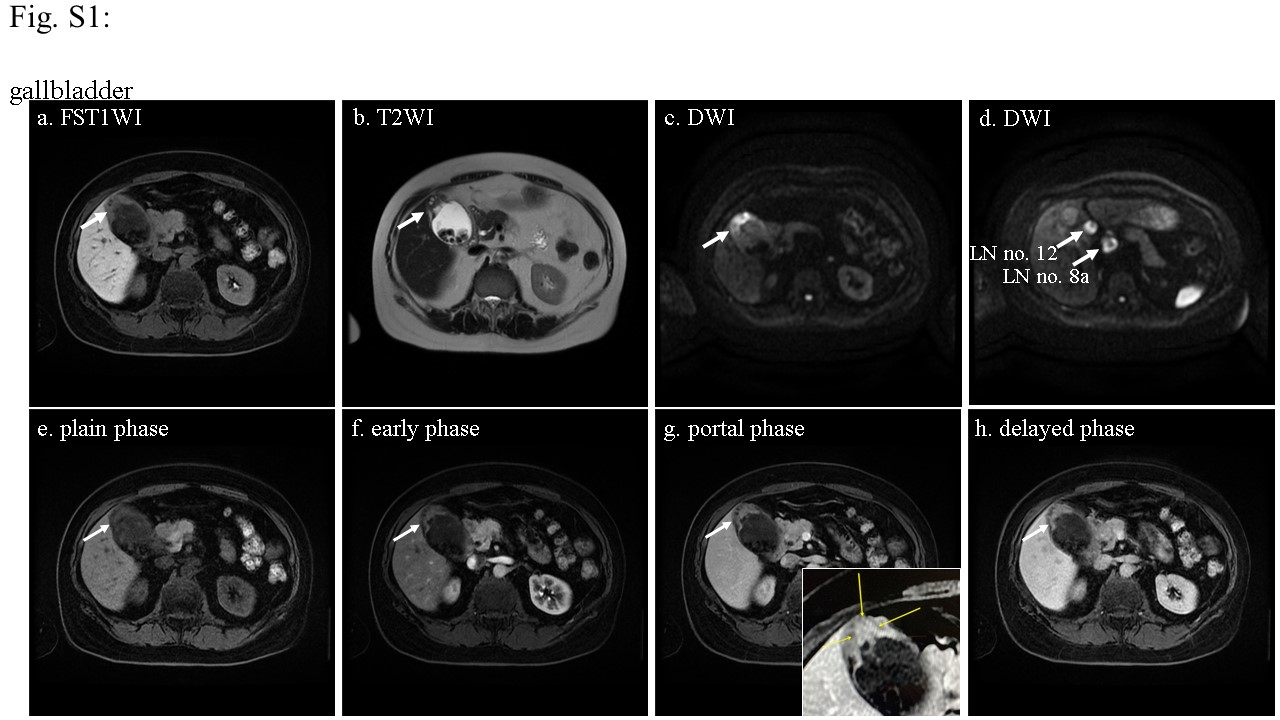

Supplement: Supplementary file 2 — Supplementary file2 Fig. S1 shows dynamic EOB-MRI results at 2 weeks prior to surgery. The irregularly thickened fundus wall of the gallbladder showed iso- to low-intensity in the fat suppressed T1-weighted image (a), and low-intensity in the T2-weighted image (b). Diffusion-weighted image of the gallbladder showed hyper intensity (c), while both enlarged LNs, no. 12 and 8a (d), showed high-intensity area, surrounding a central low-intensity area, suggestive of central necrosis. Dynamic MRI showed increased intensity in the fundus of the gallbladder (e-h), enlarged in Fig. S1g. (JPG 158 KB) [file 12328_2021_1547_MOESM2_ESM.jpg]
